# Adaptations to Post-exercise Cold Water Immersion: Friend, Foe, or Futile?

**DOI:** 10.3389/fspor.2021.714148

**Published:** 2021-07-16

**Authors:** Mohammed Ihsan, Chris R. Abbiss, Robert Allan

**Affiliations:** ^1^Human Potential Translational Research Programme, Yong Loo Lin School of Medicine, National University of Singapore, Singapore, Singapore; ^2^Research and Scientific Support, Aspetar Orthopaedic and Sports Medicine Hospital, Doha, Qatar; ^3^Centre for Exercise and Sports Science Research, School of Medical and Health Sciences, Edith Cowan University, Perth, WA, Australia; ^4^School of Sport and Health Sciences, University of Central Lancashire, Preston, United Kingdom

**Keywords:** recovery, cryotherapy, hypertrophy, mitochondrial biogenesis, muscle adaptations

## Abstract

In the last decade, cold water immersion (CWI) has emerged as one of the most popular post-exercise recovery strategies utilized amongst athletes during training and competition. Following earlier research on the effects of CWI on the recovery of exercise performance and associated mechanisms, the recent focus has been on how CWI might influence adaptations to exercise. This line of enquiry stems from classical work demonstrating improved endurance and mitochondrial development in rodents exposed to repeated cold exposures. Moreover, there was strong rationale that CWI might enhance adaptations to exercise, given the discovery, and central role of peroxisome proliferator-activated receptor gamma coactivator-1α (PGC-1α) in both cold- and exercise-induced oxidative adaptations. Research on adaptations to post-exercise CWI have generally indicated a mode-dependant effect, where resistance training adaptations were diminished, whilst aerobic exercise performance seems unaffected but demonstrates premise for enhancement. However, the general suitability of CWI as a recovery modality has been the focus of considerable debate, primarily given the dampening effect on hypertrophy gains. In this mini-review, we highlight the key mechanisms surrounding CWI and endurance exercise adaptations, reiterating the potential for CWI to enhance endurance performance, with support from classical and contemporary works. This review also discusses the implications and insights (with regards to endurance and strength adaptations) gathered from recent studies examining the longer-term effects of CWI on training performance and recovery. Lastly, a periodized approach to recovery is proposed, where the use of CWI may be incorporated during competition or intensified training, whilst strategically avoiding periods following training focused on improving muscle strength or hypertrophy.

## Introduction

Cold water immersion (CWI) is a strategy aimed at enhancing recovery from strenuous exercise, typically involving the submersion up to the waist or mid-torso for ~5–20 min in temperatures between ~8 and 15°C (Versey et al., 2013; Ihsan et al., 2016; Machado et al., 2016). Following contemporary work by Vaile et al. (2008a,b,c); Vaile et al. (2011) and Peiffer et al. (2010a,b) in late-2000s investigating the effect of CWI on the recovery of physical performance, research in this area has extended to investigate a plethora of recovery outcomes including thermoregulatory response (Stephens et al., 2018), hemodynamics (Mawhinney et al., 2013; Choo et al., 2016), hormonal balance (Halson et al., 2008), skeletal muscle damage (Goodall and Howatson, 2008), and autonomic nervous function (Bastos et al., 2012; Choo et al., 2018). Complementing this growth in research, CWI has become one of the most popular post-exercise recovery strategies utilized amongst athletes during training and competition (Périard et al., 2016; Crowther et al., 2017; Murray et al., 2018; Cross et al., 2019).

Meta-analyses and experimental research in general show that CWI can beneficially influence the recovery of physical performance (Montgomery et al., 2008; Bleakley and Davison, 2010; Rowsell et al., 2011; Poppendieck et al., 2013; Tabben et al., 2018). Nevertheless, many consider the efficacy of CWI to be equivocal. The inconsistent findings of CWI hence must be acknowledged, and this is likely driven by factors such as the nature of exercise modality preceding CWI, nature of recovery variables assessed, timing between recovery assessment and completion of CWI, and the CWI protocol itself. While such complexities need to be resolved to appropriately compare study findings and interpret with context, recent discussions have revolved extensively around how the regular use of CWI for recovery might in parallel influence adaptations to exercise (Broatch et al., 2018; Malta et al., 2021). Yet, the first study to address this extends considerably back to 2006 (Yamane et al., 2006). Following a hiatus, a series of studies examining the influence of CWI on adaptation to endurance exercise emerged from independent laboratories (Ihsan et al., 2014b, 2015, 2020b; Aguiar et al., 2016; Joo et al., 2016; Allan et al., 2017, 2019, 2020; Broatch et al., 2017), which was followed up by others examining the influence of CWI on resistance training adaptations (Frohlich et al., 2014; Roberts et al., 2015; Figueiredo et al., 2016; D'Souza et al., 2018; Fyfe et al., 2019; Fuchs et al., 2020; Peake et al., 2020; Poppendieck et al., 2020). A recent meta-analytical review showed that CWI effects on exercise adaptations are mode-dependant, where resistance training adaptations were diminished, whilst aerobic exercise performance seemed unaffected (Malta et al., 2021). Alongside these publications, detailed narrative reviews on the adaptative response following regular CWI have been published within this research topic series (Petersen and Fyfe, 2021), and elsewhere (Broatch et al., 2018). Additionally, editorials, point-counterpoints, and opinion pieces (Allan and Mawhinney, 2017; McPhee and Lightfoot, 2017; Méline et al., 2017; Peake, 2017, 2020; White and Caterini, 2017; Cheng, 2018; Ihsan et al., 2020a) have been published discussing the suitability of CWI as a post-exercise recovery tool given its dampening effect on hypertrophy gains. However, potential adaptive benefits that can be harnessed from CWI following endurance exercise, or from a recovery objective are often overlooked.

In this mini-review, we have adopted an introspective approach in discussing the molecular mechanisms and rationale surrounding CWI and endurance exercise adaptations. Moreover, we review and discuss key studies which provide information on the applied scenarios where CWI can be utilized to promote physical recovery and adaptation whilst avoiding potential negative effects on hypertrophy and strength gains.

## Cold Exposure and Endurance Exercise Adaptation

Cold stimulus is a physiological stressor capable of triggering primary signals and downstream cascades implicated in exercise-induced improvements in muscle oxidative function (Ihsan et al., 2014a). Evidence for cold-induced changes in a/the mammalian oxidative profile can be derived from as early as 1960, where Hannon (1960) demonstrated robust increases in the enzyme activities of several electron transport chain components of rat gastrocnemius muscle following 3–4 weeks of cold exposure. Subsequent studies extended these initial findings by demonstrating comparable increases in muscle oxidative enzymes between cold acclimation and exercise (Hamilton and Ferguson, 1972; Harri and Valtola, 1975). More recent studies have since shown improved fatigue resistance and exercise capacity following cold adaptation, in line with molecular signatures implicating increased mitochondrial content (Schaeffer et al., 2003; Bruton et al., 2010). In summary, research within rodent models highlight cold exposure as a viable modality to enhance muscle oxidative adaptations and endurance.

Seminal work by Spielgeman's group in the late 90's generated major breakthroughs in the mechanisms underpinning mitochondrial biogenesis (Puigserver et al., 1998; Wu et al., 1999), which incidentally further supported the use of cold therapeutic strategies such as CWI. Their work investigating the mechanisms of adaptive thermogenesis led to the discovery of the transcriptional coactivator peroxisome proliferator-activated receptor gamma coactivator-1α (PGC-1α), which was found to robustly increase in response to cold exposure (3 and 12 h exposure @ 4°C) in mice brown fat and skeletal muscle, concomitant with an increase in numerous mitochondrial markers (Puigserver et al., 1998; Wu et al., 1999). Subsequent work highlighted a regulatory role for PGC-1α in mitochondrial biogenesis (Lira et al., 2010), and other oxidative adaptations such as angiogenesis through the vascular endothelial growth factor (VEGF) (Chinsomboon et al., 2009), muscle fiber type transformation (Lin et al., 2002), glucose (Wende et al., 2005), and fat metabolism (Vega et al., 2000) in cell culture and murine models. Following evidence within human exercise models demonstrating a regulatory role for PGC-1α in skeletal muscle aerobic adaptations (Pilegaard et al., 2003; Russell et al., 2003; Perry et al., 2010), there was understandably speculation that CWI might additively enhance adaptations to exercise through common mechanisms involving PGC-1α (Ihsan et al., 2014a).

## Endurance Exercise Adaptation to Post-Exercise CWI: Discrepancy Between Molecular Signaling and Exercise Performance

Whilst CWI was not conceived as a strategy specifically meant to supplement exercise adaptations, there was substantial interest in examining how recovery-based CWI might influence skeletal muscle adaptations to endurance exercise (Ihsan et al., 2014b, 2015, 2020b; Aguiar et al., 2016; Joo et al., 2016; Allan et al., 2017, 2019, 2020; Broatch et al., 2017). This line of enquiry is likely motivated by work in cell cultures and rodents demonstrating robust increases in mitochondrial markers following exercise and cold exposure with common mechanisms involving PGC-1α. Moreover, recent work in humans extends further support, where markers of mitochondrial development have been shown to be enhanced in the skeletal muscle after acute aerobic exercise in the cold compared with room temperature (Shute et al., 2018), or when post-exercise recovery was undertaken in a cold environment (Slivka et al., 2013). In agreement, CWI (10–15 min @ 8–10°C) administered independently, or following an acute bout of endurance exercise was shown to increase the mRNA of PGC-1α and VEGF (Ihsan et al., 2014b; Joo et al., 2016). Additionally, regular CWI (15 min @ 10°C) application during 4 weeks of endurance training (30 s to 8 min interval bouts @ 80–110% of peak power/velocity) has been shown to increase a variety of mitochondrial markers (mRNA and protein abundance) and upstream regulatory kinases (Ihsan et al., 2015; Aguiar et al., 2016). However, an increase in PGC-1α protein content was not consistently observed in these studies (Ihsan et al., 2015; Aguiar et al., 2016) with only Ihsan et al. (2015) reporting an CWI-mediated increase following training. Regardless, complimenting the aforementioned studies showing an increase in VEGF mRNA (Ihsan et al., 2014b; Joo et al., 2016), regular CWI (10–15 min @ 10°C) incorporated during exercise training lasting 4–12 weeks has been shown to enhance skeletal muscle microvascular function (Ihsan et al., 2020b) and increase skeletal muscle capillarity (D'Souza et al., 2018). Conversely, Broatch et al. (2017) found no effect of CWI (15 min @ 10°C) on molecular markers indicative of mitochondrial development (i.e., mRNA responses, phosphorylation status, and protein abundance) following a single sprint interval training session or following 6 weeks of sprint interval training. Factors such as subcutaneous fat, muscle mass, body surface area, and acclimation status may influence the adaptations that are driven by the magnitude of tissue temperature change, and hence partly account for such disparity in findings. Alternatively, high-intensity exercise/training as undertaken by Broatch et al. (2017) can robustly increase mitochondrial markers (Granata et al., 2016) creating a ceiling effect, resulting in diminished potential for CWI augment further adaptations. Collectively, these findings indicate that the effects of post-exercise CWI may be less pronounced following high-intensity exercises, but is able to influence molecular and structural adaptations befitting muscle oxidative function following lower intensity endurance exercise.

Although such molecular responses would expectedly improve endurance performance in the longer term, Yamane et al. (2006) reported attenuated improvements in maximal oxygen uptake and cycling time to exhaustion following regular CWI (2 × 20 min at 5°C) during 4 weeks of endurance training. The authors suggested that the decrease in muscle temperature and metabolism following cooling might have suppressed regenerative mechanisms mediated through inflammatory and heat shock proteins (HSP). This mechanism is unlikely prevalent during recovery-based CWI protocols resulting in mild to moderate decreases in tissue temperature. Indeed, typical post-exercise CWI involves 10–15°C immersion for 10–15 min, and such protocols have been shown to not influence skeletal muscle inflammatory response, HSP expression, or trafficking (Aguiar et al., 2016; Peake et al., 2017). Interestingly, HSF-1, a transcription factor for multiple HSPs, has been shown to be upregulated following regular CWI administered throughout 4 weeks of endurance training (Aguiar et al., 2016). As such, the findings demonstrated by Yamane et al. (2006) likely involve other mechanisms such as increased muscle proteolysis, increased oxidative stress or lowered tissue metabolism and consequently remodeling (Fu et al., 1997; Manfredi et al., 2013; Broatch et al., 2018) that are associated with aggressive cooling and extreme decreases in tissue temperature.

In contrast to Yamane et al. (2006) initial report, studies investigating the longer-term effects of recovery-based CWI do not support concerns of this modality being detrimental to endurance training adaptations. For instance, regular CWI administered during 4–6 weeks of sprint- or aerobic-interval training similarly improved maximal oxygen uptake, peak aerobic power, and time-trial performance compared to control conditions (Aguiar et al., 2016; Broatch et al., 2017). Likewise, CWI administered to competitive cyclists undergoing 3-4 weeks of intensified training reported similar improvements in most cycling performance parameters, although some parameters were reported to improve to a greater extent following CWI (Halson et al., 2014).

While these findings refute suggestions that CWI might counteract endurance adaptations, it nevertheless questions whether post-exercise CWI is an effective strategy to promote muscle adaptations resulting in improved exercise performance. Indeed, changes in acute signaling response (Ihsan et al., 2014b; Joo et al., 2016; Broatch et al., 2017), training-induced protein accretion (Ihsan et al., 2015; Aguiar et al., 2016), and vascular adaptations (D'Souza et al., 2018; Ihsan et al., 2020b) do not seem to translate into improved exercise performance following regular CWI. Some have reasoned that endurance performances are largely governed by central factors (e.g., cardiovascular, hematological adaptations), and changes in muscle aerobic function following CWI may only marginally contribute (Malta et al., 2021). Alternatively, frequent CWI might have de-sensitized transcriptional responses. For instance, the magnitude of PGC-1α mRNA increases have been shown to progressively diminish in response to repeated exercise stimulus (Perry et al., 2010). Similarly, PGC-1α mRNA has been shown to robustly increase following exercise in a cold environment, but demonstrated a blunted PGC-1α mRNA response to an identical stimulus following 3 weeks of endurance training in the cold (Shute et al., 2020). However, it remains to be ascertained if this attenuated response is due to habituation to cold, exercise or a combination of both stimuli. Regardless, it must be re-iterated that CWI does not appear to impair aerobic training adaptations, and can be confidently incorporated as a recovery modality following endurance training sessions.

## Effect of CWI On Endurance and Resistance Exercise Adaptations: Divergent Effects or Coordinated Regulation?

While some studies have shown that CWI can enhance physical recovery following resistance exercise (Vaile et al., 2008c; Roberts et al., 2014), practitioners should avoid scheduling this modality at least during the immediate recovery period. Indeed, regular CWI has been shown to attenuate the magnitude of anabolic signaling (Roberts et al., 2015) and protein synthesis (Fuchs et al., 2020), leading to reduced magnitude of strength and muscle mass gain following resistance training (Frohlich et al., 2014; Roberts et al., 2015; Fyfe et al., 2019; Poppendieck et al., 2020). Readers are directed to excellent reviews elsewhere (Broatch et al., 2018; Malta et al., 2021) and within this research topic (Petersen and Fyfe, 2021) elaborating on the mechanisms surrounding CWI and resistance training.

Complimenting these mechanisms, we suggest that the attenuated increase in muscle mass observed following CWI and resistance training may be part of a macro-level mechanism protecting the oxidative profile of the muscle. This is supported by D'Souza et al. (2018) demonstrating increased muscle capillarity following 12 weeks of resistance training with regular CWI application with concomitant decreases in muscle mass reported in other companion papers (Roberts et al., 2015; Peake et al., 2017). Reductions in muscle blood flow and metabolism during CWI may reduce O_2_ supply and utilization (Ihsan et al., 2013; Mawhinney et al., 2013, 2020; Choo et al., 2016), triggering compensatory adaptation involving decreased muscle mass and microvascular expansion to maintain perfusion capacity. Further support for such a phenotypic response can be derived from rodent and human models of cold-acclimation. Cross-sectional areas of gastrocnemius and soleus muscle fibers were found to be 15–21% smaller in cold-acclimated rats, with concomitant increases in capillarity (Suzuki et al., 1997). Similarly, Bae et al. (2003) showed that cold-acclimated breath-hold divers possessed higher skeletal muscle capillary density, a lower oxygen diffusion distance and a smaller muscle fiber CSA, whist no such adaptations were evident in breath-hold divers who dived at moderate water temperatures (29–30°C) (Park et al., 2005).

While we rationalize that the dampened increase in muscle mass observed following CWI is a compensatory mechanism improving oxidative function, further research is needed to understand how this might influence athletic function and performance. For instance, it is currently unknown if CWI influences the regulation of muscle mass following aerobic exercise, and whether this hypothetical trade-off involving the attenuated increase in muscle mass and strength might be beneficial to endurance performance. On the other hand, co-assessment of muscle aerobic function within these resistance training studies (Frohlich et al., 2014; Roberts et al., 2015; Fyfe et al., 2019; Poppendieck et al., 2020) would have furthered our understanding of the functional consequence of the dampened increase in mass coupled with increased capillarity.

## CWI and Resistance Training: Insights From Applied Research

Athletes embark on a variety of training sessions such as cardiovascular conditioning, strength/resistance training, technical, and tactical work. In sports science practice, CWI is likely to be incorporated at various instances to promote recovery, particularly when recovery time between sessions is limited. Caution should be warranted against the regular use of CWI particularly following resistance exercise sessions.

Recent work (Table 1) examining the longer-term effects of CWI on training performance and recovery amongst professional and semi-professional athletes provides invaluable insights regarding CWI programming and recovery-adaptation interaction throughout training/competition phases (Lindsay et al., 2016; Tavares et al., 2019, 2020; Seco-Calvo et al., 2020). These studies collectively demonstrate no impairments in strength gains despite administering frequent post-exercise CWI over 2.5 weeks to 8 months. In contrast to the current literature (Frohlich et al., 2014; Roberts et al., 2015; Fyfe et al., 2019; Poppendieck et al., 2020), most of these studies (Table 1) report a trend for improved strength gains over the training/study period. Such divergent findings are hard to reconcile. One possibility, as Broatch et al. (2018) highlighted is that laboratory-based experimental studies are designed with 2–3 sessions per week permitting adequate recovery between sessions, and by extension not capitalizing on the recovery effects of CWI.

**Table 1:** Summary of studies examining the longer-term effect of CWI on the recovery of exercise performance.

**Study**	**Level/Sport**	**Training Phase/Duration**	**Training description**	**CWI frequency and protocol**	**CWI Timing**	**Main outcomes**
Lindsay et al., 2016	Semi-professional MMA athletes	6-week pre-competition training camp	Strength and conditioning (60–90 min, 3x/week), MMA, wrestling, jiu-jitsu, and boxing (90–120 min, 7x/week)	3x/week whole body CWI @ 10°C for 15 min	Performed following last session of the day which consisted of MMA or wresting training	Similar improvements in SBJ, pull-ups, and press-ups in CON vs. CWI group
Tavares et al., 2019	Elite Rugby Union	3 weeks during pre-season period	Strength sessions (4x/week), technical/tactical sessions (7x/week), speed, and conditioning (5x/week)	4x/week whole body CWI @ 10°C for 10 min	Performed following afternoon sessions which consisted of technical/tactical or conditioning	Better maintenance in CMJ performance in CWI group
Seco-Calvo et al., 2020	Professional Basketball players from the Spanish Premier League	Competitive season (8 months)	Gym sessions (4x/week), conditioning (3x/week), speed, and reaction (2x/week)	4x/week whole body CWI @ 10°C for 5 × 2 min	Performed following the speed and conditioning or following match-play	Better maintenance of shoulder strength
Tavares et al., 2020	U21 Portuguese national players	2.5-week pre-competition training camp	10 resistance training and 19 on-court sessions over 2.5 weeks	CWI @ 10°C for 10 min after last training session	Performed following on-court volleyball sessions	Better maintenance in CMJ performance in CWI group

*MMA, mixed martial arts; SBJ, standing broad jump; CMJ, counter-movement jump*.

Training frequency reported within these applied studies surmounts to at least 10 sessions per week (Table 1). Perhaps, in scenarios where recovery between training sessions may be limited, CWI can improve training performances and consequently the stimulus for adaptation. In support, post-exercise CWI has been shown to enhance the ability to perform more volitional work during subsequent squat exercise (Roberts et al., 2014), or better maintain day-to-day exercise performance during intensified periods of endurance training (Vaile et al., 2008b; Stanley et al., 2013). Given that adaptations to exercise stimulus are volume and intensity dependant, it seems reasonable to consider that the recovery benefits of CWI (and resultant increase in training quality) might outweigh its dampening effects on hypertrophy response. Conversely, it can be argued that anabolic adaptations are better enhanced if CWI is avoided, albeit this might deter the quality of subsequent training sessions. It is currently unknown which approach would better influence athletes' recovery-adaptation interaction. Longer term applied studies similar to those highlighted in this review (Table 1) will significantly contribute to our understanding in this area.

Another key feature of these studies (Table 1), and perhaps within sport science practice is that CWI is often not administered immediately following a resistance training session, but instead following technical/tactical or conditioning sessions. These findings show promise that beneficial recovery outcomes can be harnessed whilst avoiding negative effects of CWI on strength gains by simply avoiding the use of this recovery modality in the proximity of resistance training sessions. Regardless, we acknowledge that these studies were not specifically designed to address this notion. Moreover, the majority of these studies were relatively short-term (2.5–6 weeks). Specific, longer-term studies are therefore required to address the effect of CWI timing on strength adaptation.

## Summary and Perspectives

Cold water immersion is widely utilized by athletes during training and competition. Given that both a cold stimulus and exercise are independent stressors capable of enhancing muscle oxidative function, there remains substantial interest in examining how this modality might influence adaptations to exercise. Although post-exercise CWI up-regulates mitochondrial-related signaling, longer-term changes in protein content and result in vascular adaptations, these changes do not seem to translate to improved endurance performance. As such, further research is required to elucidate how endurance performance can be improved through its positive molecular signaling outcomes for CWI to be incorporated to enhance exercise-induced oxidative adaptations. It must be re-iterated that CWI does not impair aerobic training adaptations, and can be incorporated as a recovery modality following endurance training if needed. In contrast, regular CWI recovery incorporated into a resistance training program will dampen strength adaptations, and therefore the use of this modality following resistance exercise sessions should be discouraged. However, there is emerging data showing no impairments in strength gains in athletes incorporating regular use of CWI during intensified training periods; this either indicates that the recovery benefits conferred by CWI may outweigh its dampening effects on hypertrophy response, or the negative effects of CWI on strength may be circumvented by programing CWI following technical or aerobic conditioning sessions. In this regard, “recovery periodization” may be an important approach, where the use of CWI may be incorporated during competition or intensified training, whilst strategically avoided following training focused on improving muscle strength or hypertrophy.

## Author Contributions

All authors listed have made a substantial, direct and intellectual contribution to the work, and approved it for publication.

## Conflict of Interest

The authors declare that the research was conducted in the absence of any commercial or financial relationships that could be construed as a potential conflict of interest.
